# Cellular senescence in Crohn’s disease: a double-edged sword in intestinal fibrosis

**DOI:** 10.3389/fimmu.2026.1825690

**Published:** 2026-06-12

**Authors:** Jinguo Liu, Liangliang Yu

**Affiliations:** 1 Department of Endoscopy Center, Sir Run Run Shaw Hospital, Zhejiang University, Hangzhou, China; 2 Department of Gastroenterology, Sir Run Run Shaw Hospital, Zhejiang University, Hangzhou, China

**Keywords:** anti-fibrotic therapy, cellular senescence, Crohn’s disease, intestinal fibrosis, SASP

## Abstract

Cellular senescence represents a pivotal yet paradoxical determinant in the pathogenesis of intestinal fibrosis associated with Crohn’s disease (CD). While acute senescence may facilitate tissue repair and limit fibrogenesis, the chronic accumulation of senescent cells drives persistent inflammation and excessive extracellular matrix (ECM) deposition, ultimately leading to irreversible fibrotic strictures. This review systematically delineates the triggers of cellular senescence within the CD microenvironment-including oxidative stress, telomere dysfunction, endoplasmic reticulum stress, and genotoxic damage-and elucidates the roles of diverse senescent cell types (myo-/fibroblasts, endothelial, epithelial, and immune cells) in fibrotic progression. Central to this process is the senescence-associated secretory phenotype (SASP), which acts as a core mediator linking senescence to fibrosis through paracrine activation, ECM imbalance, immune modulation, and stem cell dysfunction. We further discuss emerging therapeutic strategies targeting senescent cells, such as senolytics and senomorphics, and highlight the translational potential of senescence-directed interventions in mitigating intestinal fibrosis. Understanding the dual roles of cellular senescence offers novel insights into the mechanisms of CD-related fibrogenesis and paves the way for innovative antifibrotic therapies.

## 1 Introduction

Crohn’s disease (CD) is a chronic, progressive inflammatory disorder of the gastrointestinal tract with rising global prevalence (1, 2). Intestinal fibrosis, manifesting as stricturing complications, develops in 30–50% of CD patients within a decade of diagnosis (3). Historically viewed as a consequence of chronic inflammation and aberrant tissue repair (4), fibrosis in CD may eventually evolve independently of inflammatory activity (5, 6). The fibrogenic process is multifactorial, involving crosstalk among immune and non−immune cells, soluble mediators, luminal microbiota, and environmental factors (7). Although therapeutic goals have advanced from symptom control to mucosal and transmural healing, current anti−inflammatory agents (including biologics and small−molecule drugs) show limited efficacy against established fibrosis. Consequently, up to 70% of CD patients require surgical intervention for fibrotic strictures, underscoring the urgent need for specific antifibrotic therapies (8).

Inflammation and fibrosis are consistent hallmarks of senescence, observed across diverse cell types and with advancing age (9). Aging stands as the primary risk factor for mortality from all major chronic diseases in adults (10). Cellular senescence-a state of stable cell−cycle arrest triggered by stress or damage-has emerged as a critical player in aging, fibrosis, and numerous chronic diseases (11–15). First described by Leonard Hayflick and colleagues in 1961 (16), cell senescence is characterized by markers such as p16^Ink4a^/p21^CIP1^ upregulation, senescence−associated β−galactosidase (SA−β−Gal) activity, and the secretion of a complex mixture of cytokines, chemokines, growth factors, and proteases termed the senescence−associated secretory phenotype (SASP) (11–13, 15, 17–19). Cell senescence participates in diverse pathophysiological processes, including telomere attrition, genomic instability, and organelle dysfunction (10, 20, 21). Our understanding, however, remains limited regarding the selective pressures exerted by aging and their impact on the functional fitness of individual organs.

Aging itself is a major risk factor for fibrotic disorders (22). Gut aging, characterized by mucosal atrophy and microbial dysbiosis, impairs intestinal barrier and immune function, predisposing to CD and other age−related pathologies (23, 24).

Cell senescence exhibits context−dependent roles: acute induction can promote wound healing and limit fibrosis (25–28), whereas the chronic persistence of senescent cells drives tissue degeneration and fibrotic progression (29). Acute senescence refers to a process in which a sudden, well-defined event triggers the cellular senescence program. Over time, the resulting senescent cells mature and are subsequently cleared by the immune system. Cells that respond acutely to damage retain the capacity to undergo apoptosis, repair, or senescence. It is important to distinguish early damage−responding cells from true senescent cells. The key distinction between them is that true senescent cells have acquired resistance to apoptosis, whereas early damage−responding cells have not. Evidence suggests that cellular senescence may contribute to fibrosis resolution (30). Supporting this, during the resolution of liver fibrosis, the numbers of both activated and senescent stellate cells decline (25, 31). However, this beneficial clearance mechanism appears impaired with age. Older organisms exhibit a diminished capacity for wound healing (32) and fibrosis resolution (33), which may explain the progression toward irreversible tissue scarring and organ damage. In CD, a disease often spanning decades, cell senescence likely contributes to the fibrotic phase. However, the precise interplay between cellular senescence and intestinal fibrosis remains inadequately defined. This review aims to systematically elucidate the mechanism of cellular senescence in the occurrence and development of intestinal fibrosis, and discuss its translational prospects as a new therapeutic target.

## 2 Drivers of cellular senescence in the CD microenvironment

Cellular senescence in CD is driven by a variety of intrinsic and extrinsic insults. Primary senescence can be triggered by oxidative damage, mitochondrial dysfunction, critical telomere shortening, genotoxic stress, oncogenic signaling, viral or bacterial infections, nutrient imbalance, and mechanical stress (12). Furthermore, secondary or paracrine senescence is often induced by extracellular inflammatory and fibrotic mediators such as TGF-β, IL-1β, IL-6, IL-8, and CCL2 (27).

### 2.1 Oxidative stress

Oxidative stress (OS) is a well-established contributor to tissue fibrosis and aging, the latter being a major risk factor for fibrotic disorders across multiple organ systems (22). Since its formal conceptualization in 1985, OS has remained a central theme in redox biology and clinical research (34). It represents a pathological state wherein pro-oxidant forces overwhelm antioxidant defenses, resulting in excessive generation of reactive oxygen species (ROS) and related oxidative intermediates (35). This imbalance manifests through two interconnected outcomes: disruption of redox-sensitive signaling pathways and accumulation of molecular damage (34, 36).

Pathological mitochondrial hyperactivation disrupts redox equilibrium: when ROS overproduction overwhelms endogenous antioxidant systems, it initiates cascading oxidative injuries, including mitochondrial DNA mutations, membrane potential collapse, and permeability transition pore opening-events central to cellular dysfunction (37). ROS specifically impairs autophagic degradation and further promotes cellular senescence (38). During chronic autophagy, ROS fuels senescence activation and myofibroblast differentiation in specific cellular subsets (39). Mechanistically, CD progression has been linked to both ROS overaccumulation and diminished antioxidant defenses (40, 41). Given that CD is a chronic inflammatory gastrointestinal disease frequently complicated by intestinal fibrosis, ROS derived from persistent intestinal inflammation is considered a principal factor driving both fibrotic transformation and cellular aging.

### 2.2 Telomere dysfunction

Telomeres critically influence cell fate and aging by modulating cellular responses to stress and mitogenic signals in the context of prior cell divisions and DNA damage (42). Chronic inflammation accelerates aging through ROS-mediated exacerbation of telomere dysfunction and cellular senescence, even in the absence of other genetic or environmental triggers (43). Many age-related pathologies, including aging itself, are closely associated with low-grade chronic inflammation (44, 45). Severe telomere dysfunction-induced by progressive shortening in late-generation telomerase-knockout mice-compromises tissue-specific stem and progenitor cell function, limits tissue regeneration, and accelerates aging (46). The high cellular turnover rate driven by chronic inflammation further accelerates telomere attrition. Telomere dysfunction elicits a persistent DNA damage response, which is a major inducer of cellular senescence (47).

### 2.3 Endoplasmic reticulum stress

Endoplasmic reticulum (ER) stress activates the unfolded protein response (UPR) through three principal branches: IRE1-XBP1, PERK-eIF2α, and ATF6 pathways (48). In CD patients, ER stress markers are elevated in stenotic regions compared to non-stenotic areas (49, 50), correlating with previously observed differences in local collagen deposition (51, 52). ER stress promotes senescence in epithelial cells and fibroblasts via the SIRT1-P300 signaling axis (53), while its inhibition alleviates senescent phenotypes (54, 55). The chaperone glucose-regulated protein 78 (GRP78) serves as a master regulator of ER homeostasis (56). Loss of GRP78 in alveolar type 2 epithelial cells (AEC2) induces ER stress, senescence, activation of TGF-β/SMAD signaling, and increased fibrotic susceptibility (57).

### 2.4 Genotoxic damage

Genotoxic stress can drive senescence through multiple mechanisms. For instance, stress-induced miR-494 suppresses DNA repair pathways, exacerbates DNA damage, and promotes endothelial senescence by interfering with telomerase activity (58).

Genotoxic stress also disrupts stem cell differentiation by integrating aberrant DNA damage responses (59). Persistent DNA damage may overwhelm repair systems and lead to senescence (60). Patients with CD exhibit higher levels of general and oxidative DNA damage compared to controls (61). Inflammatory mediators such as TGF-β and IL-22, along with microbial products from viral or bacterial infections, can induce DNA damage. TGF-β, for example, triggers DNA damage, mitochondrial ROS production, and ultimately senescence in adipose progenitor cells (62). Conversely, the DNA damage response can reinforce growth arrest by activating TGF-β signaling (63). In colitis-associated cancer models, IL-22 promotes nitric oxide-dependent DNA damage (64).

**Figure 1:**
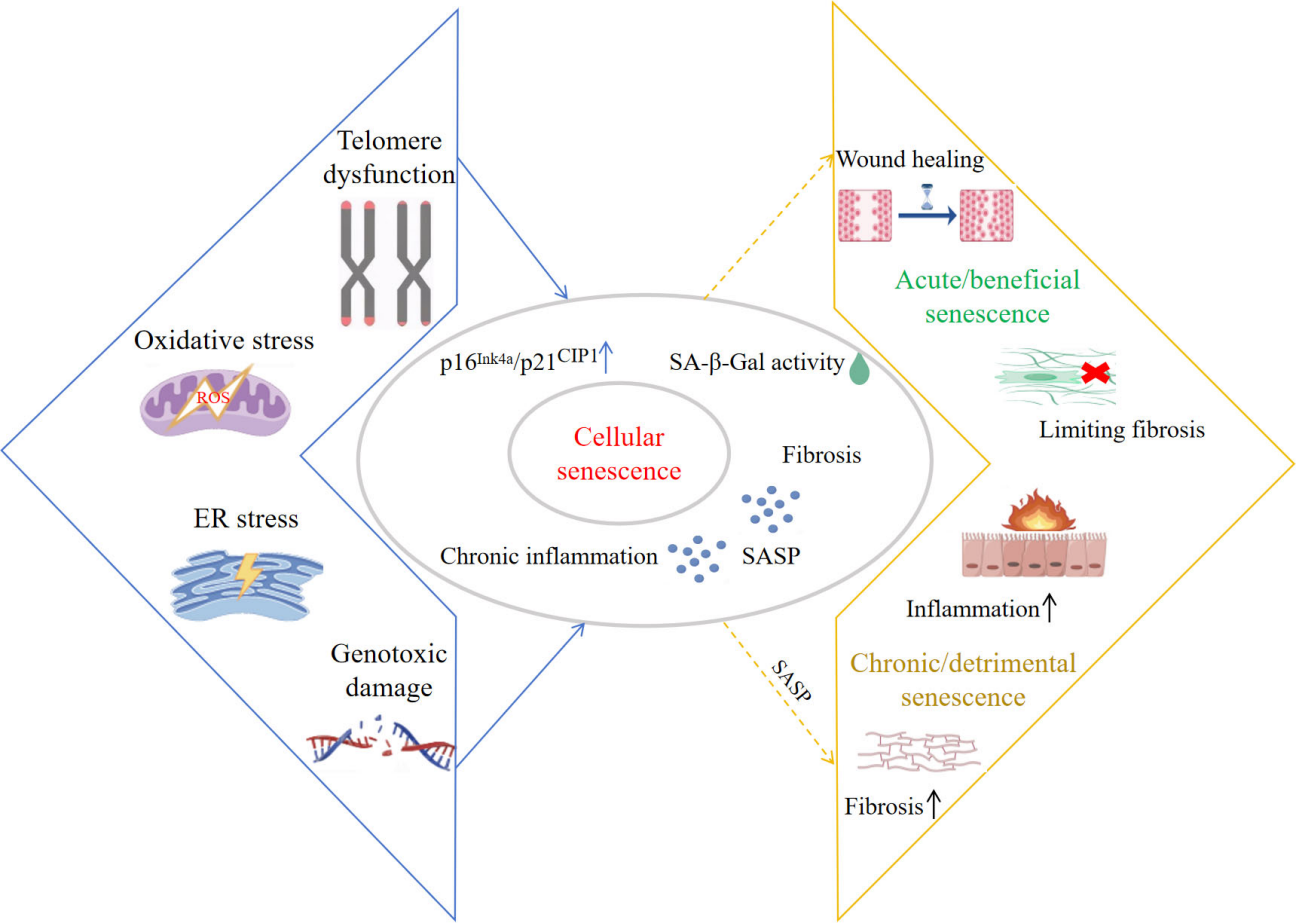
Triggers and consequences of cellular senescence. This schematic summarizes the key mechanisms linking the disease microenvironment to cellular senescence and its divergent outcomes in CD. The left panel illustrates four primary drivers: oxidative stress, telomere dysfunction, ER Stress, and genotoxic damage. These converge centrally to induce a core senescence program, marked by upregulation of p16^Ink4a^/p21^CIP1^ and other hallmarks such as SA-β-Gal activity, SASP, chronic inflammation and fibrosis. The right panel highlights the context-dependent duality of senescence: Acute/beneficial senescence promotes tissue repair and limits fibrosis, whereas chronic/detrimental senescence drives sustained inflammation and, via the SASP, propagates intestinal fibrosis.

Collectively, these drivers-oxidative stress, telomere dysfunction, ER stress, and genotoxic damage-interact within the inflammatory milieu of CD to establish and perpetuate a senescent cell burden. This senescent microenvironment in turn fosters fibrosis, impairs tissue repair, and contributes to disease progression, highlighting senescence as an integrative pathological node in CD (**Figure 1**).

## 3 Types and functions of intestinal senescent cells

Senescent cells accumulate in human tissues with advancing age, showing increases ranging from 2- to 20-fold when comparing young (<35 years) to old (>65 years) healthy individuals (65). While all cell types can undergo senescence, in the intestine, fibroblasts, macrophages, and endothelial cells are predominantly affected (66). Critically, extracellular signals from both resident and infiltrating immune cells-including macrophages, endothelial cells, and T, B, and NK lymphocytes-play pivotal roles in regulating the activation and phenotypic shift of fibroblasts, thereby shaping the fibrotic landscape (67, 68). This section delineates the specific contributions of each major cell type to the establishment and maintenance of a pro-fibrotic, senescence-rich milieu in CD.

### 3.1 Fibroblasts and myofibroblasts

In CD, fibrosis and stricture formation are driven by dysregulated extracellular matrix (ECM) deposition. Although senescent myo-/fibroblasts may exhibit diminished proliferative capacity, they remain metabolically active and potently drive ECM deposition through the sustained secretion of a profibrotic SASP, notably TGF-β1 (7). This functional shift underscores how senescence critically influences the fibrogenic behavior of myo-/fibroblasts in CD. Supporting this, myofibroblasts isolated from stenotic mucosa in CD show elevated TGF-β expression and collagen production compared with those from non−stenotic regions (69). Beyond the traditional role of myofibroblasts as the primary ECM producers, recent findings highlight specialized fibroblast subpopulations-marked by expression of CXCL14, MMP/WNT5A, and CTHRC1-as central signaling nodes within CD fibrotic strictures (70, 71). These studies reposition senescent fibroblasts not merely as matrix producers but as dynamic signaling hubs that orchestrate fibrogenesis. Their pathophysiological role is further evidenced in perianal fistulizing CD (72). The senescent fibroblast phenotype is characterized by enhanced apoptosis resistance and the hypersecretion of a broad repertoire of inflammatory cytokines, growth factors, immune modulators, and proteases, thereby perpetuating a self-sustaining cycle of inflammation and fibrosis (73, 74). In complicated ileal CD, this apoptotic resistance correlates with a distinct inflammatory and fibrogenic expression profile, including the induction of both TGF-β1 and IL-1β, effectively replicating a local SASP (75). The induction of fibroblast senescence is context-dependent, triggered by factors such as mechanical stress (76) or systemic mediators like visceral adipose-derived osteopontin (77). Interestingly, the induction of senescence can serve a paradoxical, beneficial role in certain contexts. For instance, the matricellular protein CCN1 induces fibroblast senescence to restrict fibrosis during cutaneous wound healing (78). This evidence suggests a dual nature for myofibroblast senescence-acting as an initial antifibrotic brake, as seen in myocardial fibrosis where it is a therapeutic target (79), but, when chronic, becoming a maladaptive driver of persistent ECM deposition.

### 3.2 Endothelial cells

Endothelial cell senescence disrupts vascular homeostasis and fosters a pro-fibrotic microenvironment. A key mechanism linking endothelial senescence to fibrosis in CD is endothelial-to-mesenchymal transition (EndoMT). Chronic inflammation drives this process, wherein intestinal microvascular endothelial cells transdifferentiate into mesenchymal cells under the synergistic influence of TGF-β1, IL-1β, and TNF-α (80). Thus, endothelial senescence and EndoMT represent convergent pathways through which chronic inflammation directly fuels tissue fibrosis. Chronically elevated TNF-α is a potent inducer of premature endothelial senescence, contributing to the vascular pathology of chronic inflammatory diseases (81). The functional significance of senescent endothelial cells is underscored by interventional studies: eliminating p16^Ink4a+^ endothelial cells aggravates liver injury, while their targeted reprogramming attenuates fibrosis, highlighting their active role in disease progression (82). Over time, sustained endothelial senescence leads to progressive structural and functional deterioration of the vasculature (83).

### 3.3 Epithelial cells

Senescence of intestinal epithelial cells compromises barrier integrity and actively shapes the local microenvironment via SASP factors. This is highly relevant to CD fibrotic stenosis, where expansion of mesenchymal cells and excessive ECM deposition-processes often linked to epithelial-mesenchymal transition (EMT)-are hallmark features (6, 51). The presence of senescent (p16^+^, p21^+^) epithelial cells within the intestinal stem and progenitor cell niches in CD suggests a direct impact on tissue regeneration and homeostasis (84). The finding positions epithelial senescence as an upstream event that may disrupt mucosal repair and initiate pro-fibrotic signaling. Parallels from pulmonary fibrosis robustly support the causative role of epithelial senescence: a senescence-enriched transitional AEC state persists in fibrotic lungs (85, 86), and senescence of AEC2 alone is sufficient to drive progressive fibrosis (87). Single-cell analyses in idiopathic pulmonary fibrosis have identified aberrant basaloid cell populations expressing senescence markers (88), and mechanisms such as epithelial CD38 activity have been shown to promote fibrosis by accelerating cellular aging (89). Importantly, epithelial cells also possess inherent protective mechanisms. For example, extracellular vesicles from bronchial epithelial cells can inhibit both TGF-β-induced myofibroblast differentiation and epithelial senescence (90). The profibrotic role of senescent epithelial cells extends beyond the gut, as evidenced in renal and hepatic diseases where they produce SASP components like TGF-β1 to induce fibrosis (91, 92).

### 3.4 Macrophages

Immunosenescence, defined as the age-related decline in immune system function, encompasses several hallmark features: thymic involution, hematopoietic stem cell (HSC) dysfunction, disruption of the naïve-to-memory ratio in T and B cells, inflammaging, accumulation of senescent cells, impaired responses to neoantigens, mitochondrial dysfunction, genomic instability, and dysregulated stress responses (93, 94). Immunosenescence alters macrophage function, exacerbating immune dysregulation and fibrosis. Senescent fibroblasts can stimulate immunosuppressive M2 macrophages, which in turn promote excessive fibroblast-to-myofibroblast conversion, creating a feed-forward loop that aggravates tissue fibrosis (95). This crosstalk establishes a vicious cycle where senescent cells modulate the immune landscape to favor fibrogenesis. A defined pathological circuit involves pro-inflammatory macrophages (FCN1^+^IL1B^+^) inducing inflammation-associated fibroblasts, which then produce the key fibrogenic cytokine IL-11 (96). M2 macrophages, as key mediators of chronic inflammation, further interact with mesenchymal stromal cells via paracrine mechanisms to suppress their antifibrotic functions (97). Such specific fibroblast-macrophage interactions are also implicated in age-related fibrosis in other organs, like the ovary (98). In liver fibrosis, senescent macrophages constitute a distinct pathogenic population, and their clearance (p16^Ink4a+^) significantly mitigates tissue damage (82). Profibrotic macrophage subsets are a consistent feature across fibrotic diseases, as seen in pulmonary fibrosis (88). Targeting these cells is therapeutically promising, as demonstrated by the inhibition of the metabolic enzyme ACSS2, which alleviates IL-1β-driven macrophage activation and tubular cell senescence to delay renal fibrosis (99).

### 3.5 T lymphocytes

Aging and chronic inflammation lead to the accumulation of dysfunctional, terminally differentiated senescent T cells. These cells secrete abundant proinflammatory factors, contributing to immunosenescence (100). In the context of CD, persistent antigen exposure in the chronically inflamed gut drives CD4^+^ and CD8^+^ T cell activation, exhaustion, and terminal differentiation, accompanied by a relative enrichment of regulatory T (Treg) cells (101). This altered T cell repertoire reflects and reinforces the chronic inflammatory milieu that predisposes to fibrosis. However, T cell exhaustion is distinct from immunosenescence. For instance, CD8+ T cells expressing PD-1 or TIM-3 are more accurately characterized as exhausted-exhibiting reversible dysfunction and retained proliferative capacity upon checkpoint blockade (102, 103), rather than senescent. Although activated immune cells express senescence markers in certain situations, they are not necessarily senescent, as they retain the capacity to proliferate upon stimulation. Replicative senescence in T cells is mechanistically driven by repeated stimulation-induced telomere attrition and DNA damage (104, 105). FOXP3^+^ Treg cells, involved in maintaining immune tolerance, are considered a potential therapeutic target in CD (106). Notably, intrinsic alterations in CD4**^+^** T cells are sufficient to drive chronic inflammation and accelerate a systemic aging phenotype (107), linking adaptive immunity directly to organismal aging processes. Furthermore, the presence of highly differentiated CD8^+^ T cells with markers of DNA damage (γ-H2AX+) and shortened telomeres is associated with severe liver fibrosis (108), indicating a potential circulating biomarker role for senescent T cells in fibrotic diseases.

### 3.6 B lymphocytes

Emerging evidence highlights a significant role for B lymphocytes in modulating fibrosis resolution. In mouse models of liver injury, B cells actively limit the resolution of senescence-driven fibrosis (109), suggesting an inhibitory role in tissue repair. This effect can be therapeutically targeted, as demonstrated by mesenchymal stem cells which alleviate liver fibrosis partly by suppressing the proliferation and cytokine production of intrahepatic B cells (110). These findings reveal B cells as active regulators of the fibrotic niche rather than passive bystanders. Further supporting this, pharmacological strategies like B-cell lymphoma-extra large (BCL-xL) inhibitors, which target senescent cholangiocytes and activated fibroblasts, have shown efficacy in ameliorating fibrosis in mice (111).

### 3.7 NK cells

Aging reshapes the natural killer (NK) cell compartment, driving a redistribution of subsets characterized by an increase in mature NK cells and a marked decline in immature populations, likely attributable to diminished bone marrow precursor output in the elderly (112, 113). This shift may influence immune surveillance in age-related pathologies. In the context of CD, pro-inflammatory NK-like T cells are expanded within the inflamed intestinal mucosa (101), suggesting a role in local immune activation. Notably, senescent cells can evade NK cell-mediated immune clearance by upregulating surface expression of the disialylated ganglioside GD3 (114). This immune evasion mechanism allows senescent cells to persist and exert their deleterious effects. When functionally competent, NK cells are crucial for resolving fibrosis, as they preferentially eliminate senescent activated stellate cells in liver injury (25). This vital antifibrotic function is compromised in idiopathic pulmonary fibrosis (IPF), where both the proportion and cytotoxic activity of NK cells are reduced. Critically, the IPF lung microenvironment itself induces a dysfunctional NK cell phenotype, creating a permissive environment for senescent cell accumulation and hindering fibrosis resolution (115).

In summary, cellular senescence in CD is not a cell-autonomous phenomenon but a network pathology. Fibroblasts, myofibroblasts, endothelial, epithelial, and immune cells (macrophages, T, B, and NK lymphocytes) all undergo senescence and engage in extensive crosstalk via the SASP and direct contact. This interplay creates a self-perpetuating cycle that sustains chronic inflammation, disrupts tissue architecture, and promotes progressive fibrosis. Understanding the unique and interconnected contributions of each cellular player within this network is fundamental for developing targeted therapies, such as senolytics or senomorphics, aimed at disrupting this pathogenic circuit and altering the course of CD.

## 4 SASP: the core bridge connecting cellular senescence and intestinal fibrosis

**Figure 2:**
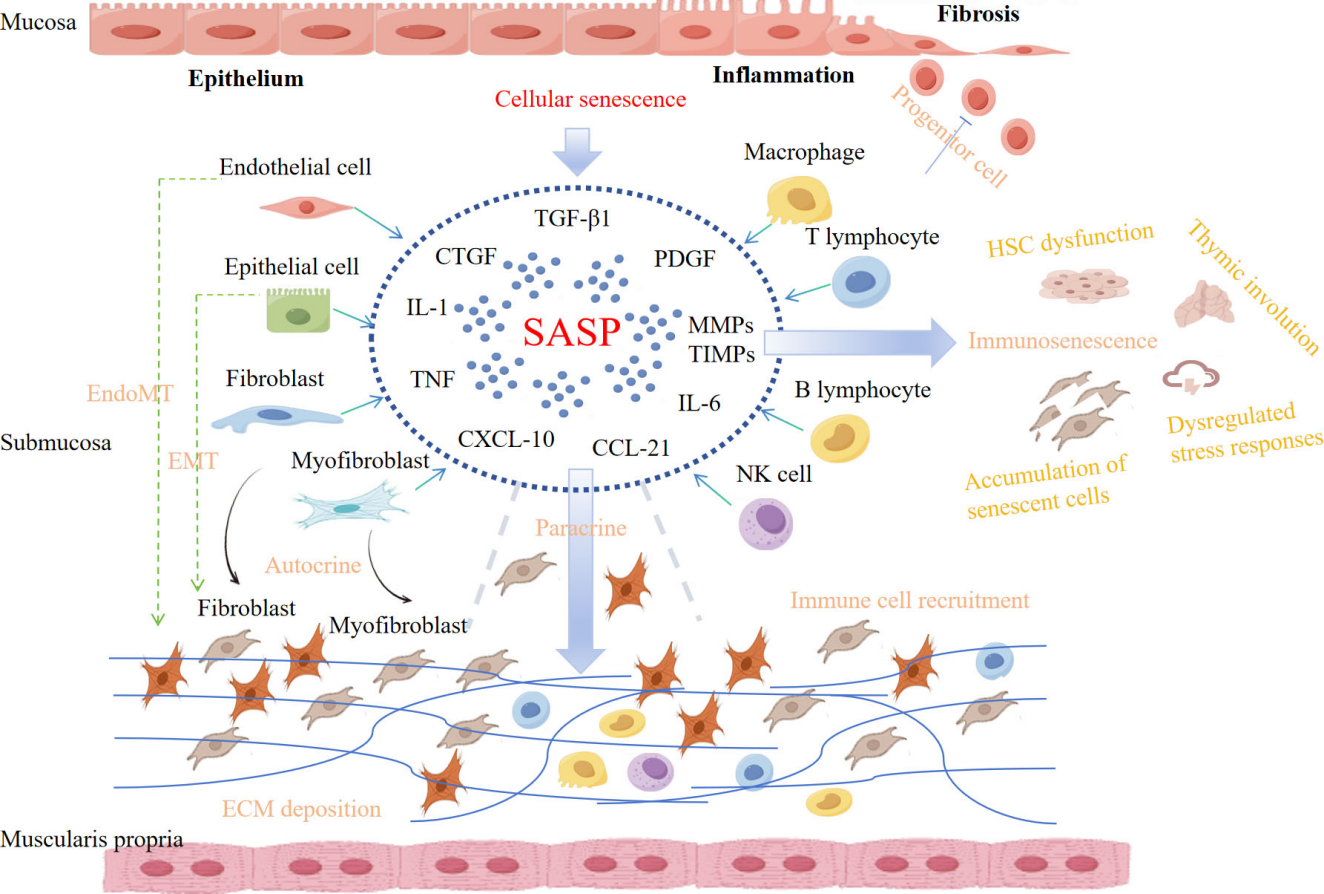
SASP-mediated crosstalk in intestinal fibrosis. This illustration delineates the central role of SASP as the core orchestrator of a cellular network during the evolution from normal intestinal epithelium to inflammation and fibrosis. SASP is centrally positioned as a dynamic signaling program, wherein senescent cell populations secrete a plethora of mediators, including TGF-β1, IL-6, IL-1, CXCL-10, and MMPs/TIMPs. These populations include senescent fibroblasts/myofibroblasts (paracrine/autocrine activation and enhanced ECM production), senescent endothelial cells (EndoMT), senescent epithelial cells (EMT), and senescent immune cells (immune dysregulation and impaired clearance). Senescent immune cells secrete abundant proinflammatory factors, contributing to immunosenescence, which mainly includes thymic involution, HSC dysfunction, accumulation of senescent cells, and dysregulated stress responses. Through these distinct yet interconnected pathways, the SASP-driven dysfunctions converge to fuel the pathological endpoint of intestinal fibrosis in the submucosa, characterized by excessive ECM deposition.

SASP constitutes the principal mechanism through which senescent cells exert their local and systemic effects. In the context of intestinal fibrosis, the SASP acts as a critical signaling hub, transmitting pro-fibrotic signals that disrupt tissue homeostasis and drive pathological remodeling (**Figure 2**).

### 4.1 The pro-fibrotic components of SASP

During intestinal inflammation, mesenchymal cells reside within a complex microenvironment replete with luminal antigens and a diverse array of inflammatory mediators released by both immune and non-immune cells (7). The SASP significantly enriches this milieu, with its core pro-fibrotic cytokines including IL-1β, IL-6, and TNF (116–118). This persistent mediator-rich environment is believed to be crucial not only for initiating but also for perpetuating the cycle of intestinal fibrosis (119).

#### 4.1.1 Growth factors

##### 4.1.1.1 TGF-β1

Transforming growth factor-beta 1 (TGF-β1) is universally recognized as the master regulator of fibrosis and is upregulated across virtually all fibrotic diseases (120), including CD (121). In CD, a pronounced pro-fibrotic signaling axis is evident in strictured areas: myofibroblasts exhibit elevated TGF-β1 gene expression and phosphorylated SMAD2/3 (its positive regulators), concurrent with downregulation of the inhibitory SMAD7 (69). The centrality of TGF-β1 is further validated by interventional studies: accination against TGF-β1 attenuates fibrosis, whereas inhibition of SMAD7 exacerbates it in murine colitis models (122, 123). TGF-β1 stimulation elicits a dose-dependent fibrogenic response in human fibroblasts, upregulating α-SMA, collagen I, and fibronectin (124), with the muscularis layer showing particularly pronounced fibrogenesis compared to the mucosa (125).

##### 4.1.1.2 CTGF

Connective tissue growth factor (CTGF) operates as a critical downstream effector of TGF-β signaling (126, 127). It directly stimulates fibroblast proliferation and ECM production in CD patients (128), with its mRNA prominently localized to submucosal fibroblasts (129). Co-expression of TGF-β1, CTGF, and collagen-1alpha is a hallmark of stricturing CD tissue (130). This axis is reinforced by phospho-STAT3(S727) signaling, which drives the production of these pro-fibrotic molecules in ileal strictures (131). Furthermore, mechanical stress within the gut upregulates CTGF, implicating it in the development of fibrostenotic complications (132, 133).

##### 4.1.1.3 PDGF

Platelet-derived growth factor (PDGF) potently induces collagen secretion by intestinal fibroblasts in CD (134). A pro-fibrotic network is evident in creeping fat, where preadipocytes exhibit enhanced communication via PDGF and other growth factors (135). A distinct pathogenic mechanism involves IgG4-producing plasmablasts, which secrete PDGF to activate PDGF-receptor-expressing fibroblasts and myofibroblasts, driving uncontrolled fibrosis in stricturing CD (136). Correspondingly, PDGF receptor beta (PDGFRB) is overexpressed in the fibrotic ileal tissues of these patients (137).

Growth factors, such as TGF-β1, CTGF, and PDGF, form an interdependent signaling network that amplifies fibrogenic responses. TGF-β1 acts as the primary initiator, CTGF translates and extends its signals, and PDGF provides potent mitogenic and activating cues to mesenchymal cells, creating a multi-layered driver of ECM deposition.

#### 4.1.2 Inflammatory cytokines

##### 4.1.2.1 IL-1 family of cytokines

Cytokines of the IL-1 family, including IL-1α, IL-1β, IL-33, and IL-36 isoforms (α, β, γ), possess significant pro-fibrotic properties (138). Epithelium-derived IL-1α is a potent fibroblast activator and can reignite intestinal inflammation (139). IL-1β is a key component of the SASP signature replicated in the ileal tissue of CD patients (75) and directly stimulates intestinal myofibroblasts to produce collagens I and IV (140). IL-33, signaling through ST2, induces EMT and fibroblast proliferation (141). IL-36 isoforms are produced by various cells, including fibroblasts and myofibroblasts (142, 143). In CD, IL-36α and γ are induced, correlating with IL-1β levels (144), and are thought to exert pro-inflammatory effects via induction of CXC chemokines (145).

##### 4.1.2.2 IL-6 family of cytokines

The IL-6 family cytokines, primarily IL-6, IL-11, and IL-27, signal through a common gp130 receptor subunit (146). IL-6, sourced from both epithelial and mesenchymal cells, is well-established in CD pathogenesis (119, 147). Its level is increased in the muscle layer of strictured CD and can be induced by TGF-β1; conversely, IL-6 neutralization normalizes TGF-β1 expression (131). Fibrosis can be targeted via IL-6-dependent mechanisms using PI3K or histone deacetylase inhibitors (148). IL-11 acts as a potent fibrogenic factor through paracrine activation of its receptor (IL-11RA) on mesenchymal cells (149, 150). It is significantly upregulated in stenotic areas of CD (151, 152), and its overexpression in fibroblasts or smooth muscle cells is sufficient to induce experimental intestinal inflammation and fibrosis (153). while IL-27 can possess anti-fibrotic properties, its inactivation by externalized histones exacerbates pulmonary fibrosis by unleashing platelet-derived TGF-β1 (154), and it promotes cardiac fibroblast activation after myocardial infarction (155).

##### 4.1.2.3 TNF family of cytokines

Tumor necrosis factor (TNF) and TL1A (TNF-like ligand 1A) drive intestinal fibrosis both indirectly by perpetuating inflammation and directly by acting on mesenchymal cells (119, 156). TNF promotes fibrosis by enhancing collagen I and IV production by myofibroblasts (140) and upregulating TGF-β and tissue inhibitor of matrix metalloproteinases 1 (TIMP1) in epithelial cells (157). While TNF inhibition can reduce collagen production and experimental fibrosis (158, 159), anti-TNF antibodies have not proven effective in preventing CD strictures clinically (156). TL1A emerges as a promising target with its high peripheral expression correlating with stricturing behavior in CD (160). It directly signals to intestinal myofibroblasts to increase collagen production, and its neutralization reduces TGF-β1 expression and myofibroblast numbers (161, 162).

These cytokine families illustrate how chronic inflammation and fibrosis are inextricably linked. They transition the tissue from a state of acute, resolvable injury to one of chronic, pathological repair, with SASP components like IL-1β, IL-6, and TL1A serving as direct molecular bridges between immune activation and mesenchymal cell dysregulation.

#### 4.1.3 Chemokines

Key SASP chemokines, such as CXCL-10, CXCL-12, CXCL-14, and CCL-21 (163, 164), orchestrate leukocyte recruitment and fibroblast activation. CXCL-10 production is enhanced in the buccal epithelium of children with CD (165), a process driven by gut microbiota-induced NF-κB activation (166), and it contributes to liver inflammation and fibrosis (167). CXCL-12, via its receptor CXCR4, directs fibrocytes to fibrotic sites (168) and promotes lung fibrosis by recruiting SMAD3 to the CTGF promoter (169). CXCL14 expression in kidney fibroblasts is upregulated by oncostatin M (170). CCL-21 modulates the properties of fibroblasts in IPF but is not universally upregulated in fibrotic lungs (171, 172).

#### 4.1.4 Matrix metalloproteinases and their inhibitors (TIMPs)

ECM accumulation results from an imbalance between its synthesis and degradation, governed by MMPs and TIMPs (173). However, the expression profile of these mediators in CD is complex and appears context-dependent. While it has been reported no significant differences in the expression of key MMPs (1–3, 9) or TIMPs (1, 2) when comparing inflamed fibrotic mucosa to inflamed non-fibrotic mucosa (174), other evidence indicates a marked increase of TIMP-1 level in full-thickness fibrotic ileal tissue from CD patients compared to controls (69, 175). These observations suggest a dynamic and phase-dependent dysregulation of the protease-antiprotease system. Initially, MMP activity may be low, favoring ECM accumulation in CD patients (173). Meanwhile, certain MMPs also have pro-fibrotic actions, such as activating latent TGF-β (MMP-2, -9) or promoting EMT (MMP-3) (176, 177). Ultimately, an imbalanced MMP9/TIMP1 ratio, with a decrease in MMP9 and an increase in TIM1, is associated with fibrotic stenosis in CD (178). In conclusion, the MMP/TIMP imbalance in fibrosis is dynamic: early stages feature increased MMP activity driving collagen fragmentation and matrix remodeling, whereas late fibrostenotic lesions exhibit TIMP overactivity that inhibits collagen degradation and promotes net accumulation of cross-linked collagen.

### 4.2 Multiple mechanisms of SASP-driven fibrosis

#### 4.2.1 Paracrine activation

The SASP modifies the tissue microenvironment by inducing paracrine senescence, modulating immune cell activity, and altering ECM dynamics, all central to fibrotic pathogenesis (179). SASP enables senescent cells to drive paracrine senescence, critically affecting neighboring cell function (180). Specifically, the SASP activates surrounding quiescent fibroblasts, stimulating them to produce excessive ECM either directly or following their differentiation into myofibroblasts. For instance, paracrine autophagy-dependent fibroblast growth factor 2 (FGF2) from injured tubular cells activates fibroblasts, contributing to renal fibrosis (181). This paracrine spread of senescence uncouples tissue injury and, by expanding the senescent cell population, further amplifies the SASP in a feed-forward manner (182). Conversely, therapeutic induction of a modified senescent secretome in hepatic stellate cells via ASCT2 inhibition can inhibit their paracrine pro-fibrotic effects (31), and compounds like andrographolide can suppress SASP-mediated fibroblast activation (183).

#### 4.2.2 Imbalanced ECM homeostasis

SASP promotes ECM synthesis such as collagen, while inhibiting ECM degradation by inhibiting MMPs and increasing TIMPs. ECM remodeling homeostasis is governed by the balance between MMPs and TIMPs (184, 185). In aging, SASP components like collagen-cleaving MMPs promote ECM degradation (186), a finding corroborated by elevated MMP-1, -2, and -9 in aged fibroblasts (187, 188). The fibrotic process is further sustained by TGF-β, which stabilizes the activated fibroblast phenotype, resulting in excessive ECM deposition and maladaptive remodeling (189). The proteolytic milieu is intensified by upregulated TNF-α and MMPs, both contributing to ECM degradation (190). A hallmark alteration is the shifted MMP/TIMP ratio, where increased MMP activity coupled with diminished TIMP inhibition culminates in progressive collagen fragmentation (191).

#### 4.2.3 Immune cell recruitment

SASP mediates pleiotropic effects in a context-dependent manner. It reinforces cell cycle arrest, recruits immune cells, and orchestrates tissue responses that can either promote repair or drive pathology (192). Beneficially, specific SASP factors recruit immune effector cells-such as NK cells (25), macrophages (193), and T cells (194)-to facilitate the clearance of senescent cells (195). Other components signal damage to neighboring tissues, stimulating repair and limiting excessive fibrosis (78, 196). Following injury, SASP factors can also enhance cellular reprogramming, plasticity, and stemness in adjacent cells to aid regeneration (197). Paradoxically, however, the SASP is a major driver of age-related tissue dysfunction. By establishing a chronic pro-inflammatory microenvironment, many SASP factors contribute to the progressive loss of tissue structure and function (27).

#### 4.2.4 Inhibition of stem/progenitor cell function

Senescent (p16^+^, p21^+^) epithelial cells in the stem and transient amplifying cell niches of the intestine are present in CD (84). Fibrosis reversal is fundamentally compromised when the regenerative potential of intestinal stem/progenitor cells is inhibited. A key amplifier of this dysfunction is cellular senescence, which not only reduces cellular functionality but also initiates the pro-inflammatory SASP-a proteome containing cytokines like IL-6, IL-1, CXCL10, and TNF-α (12). This creates an altered microenvironment where the SASP, combined with innate immune cell infiltration and inflammation stemming from stem cell dysfunction, promotes pro-fibrotic signaling-notably through TGF-β activation (198). Senescence of specific progenitor populations is a pivotal cause of lost stemness (199), and even fibroblast senescence can modulate adjacent stem cell function (200). Promising anti-fibrotic approaches aim to target this senescent niche, leveraging mesenchymal stem cells and their paracrine factors (201–203) or rejuvenating endogenous stem cells through mechanisms like autophagy enhancement (204).

SASP is not a mere bystander but the central executor of senescence-mediated fibrosis. It operates through a multi-pronged strategy: providing sustained mitogenic and activating signals via growth factors; maintaining a chronic inflammatory state via cytokines and chemokines; dysregulating ECM turnover via MMPs/TIMPs; paralyzing tissue repair by inhibiting stem cells; and perpetuating the senescent phenotype via paracrine signaling. This comprehensive assault on tissue homeostasis underscores why targeting the SASP or its producing cells represents a potential therapeutic strategy for mitigating fibrosis in CD.

## 5 Targeting cellular senescence: a novel strategy for treating intestinal fibrosis

**Figure 3:**
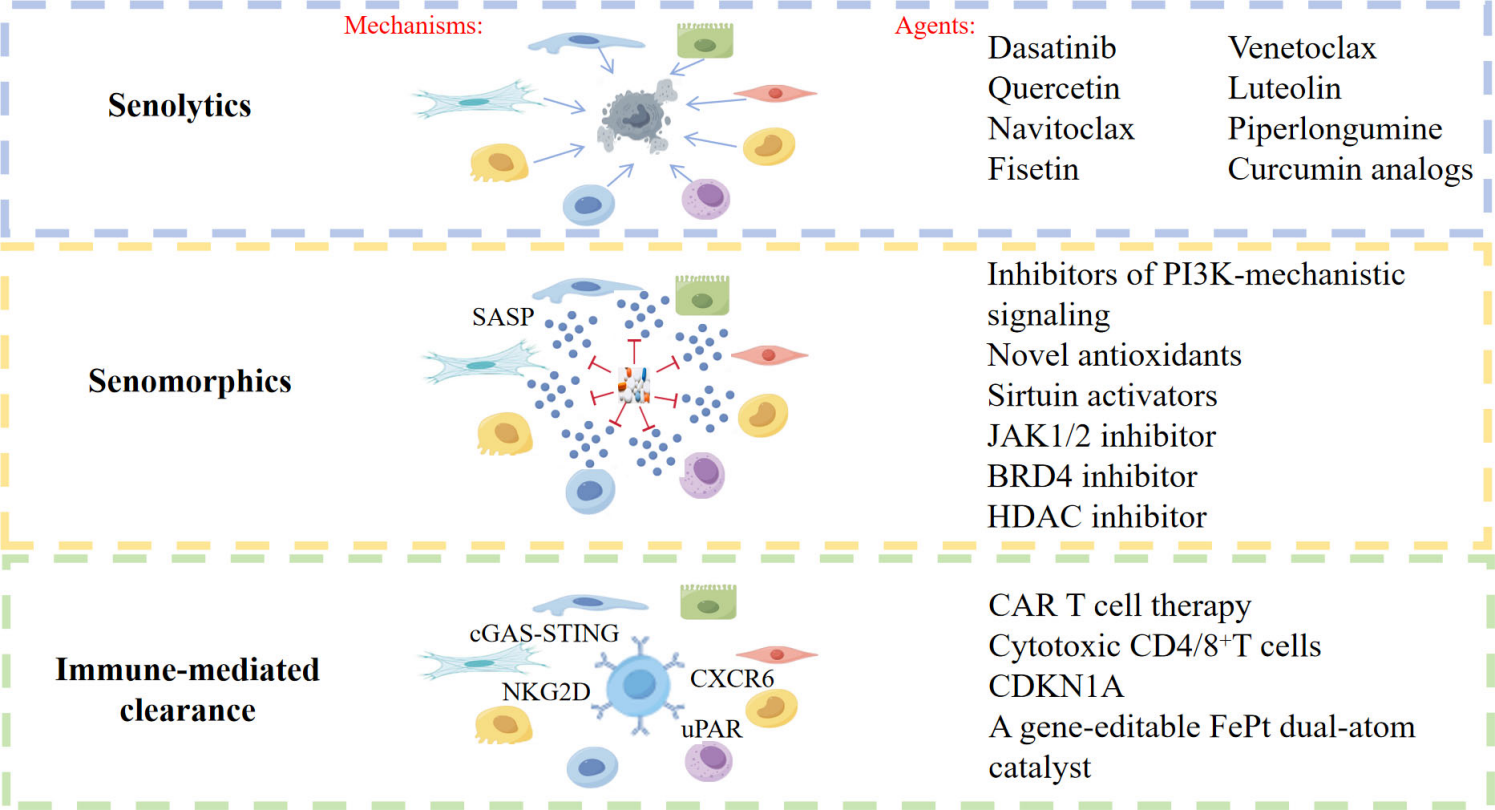
Therapeutic strategies targeting senescent cells in intestinal fibrosis. This image outlines three principal and complementary therapeutic approaches to disrupt the senescence-fibrosis axis in CD. The strategies include: Senolytics selectively eliminate senescent cells (e.g., via Dasatinib + Quercetin, Navitoclax), leading to a reduced senescent burden and attenuated fibrosis; Senomorphics modulate the senescent phenotype by suppressing the SASP (e.g., via JAK1/2 or cGAS-STING inhibition), thereby blunting paracrine fibrotic activation; Immune-Mediated Clearance employs engineered or enhanced immune effectors (e.g., CAR-T cells) to precisely target and remove pathogenic senescent cell populations, restoring tissue immune surveillance. These strategies aim to break the vicious cycle of senescence-driven fibrosis, offering potential for combination therapy with existing anti-inflammatory agents in CD.

The recognition of cellular senescence as a key driver of chronic inflammation and fibrosis has spurred the development of novel therapeutic approaches aimed at mitigating its detrimental effects. These strategies primarily fall into three categories: eliminating senescent cells (senolytics), modulating their pathological secretome (senomorphics), and enhancing their immune-mediated clearance. Each approach offers a distinct mechanism to disrupt the pro-fibrotic network sustained by senescent cells (**Figure 3**).

### 5.1 Senolytics

Senolytics, a class of drugs designed to selectively eliminate senescent cells, represent a prominent therapeutic strategy in this field. The most compelling evidence for the causal role of senescence in aging and disease stems from studies showing that genetic or pharmacological clearance of these cells extends healthspan and ameliorates various pathologies in aged mice (205, 206). The pioneering senolytics, discovered via hypothesis-driven approaches, include dasatinib, quercetin, navitoclax (ABT263), and fisetin (207). The combination of dasatinib and quercetin (D+Q) has demonstrated efficacy in reducing senescent cell burden in contexts such as hepatic steatosis in obese mice (208), improving age-related vascular dysfunction (209), and attenuating intervertebral disc degeneration (210). D+Q, along with B-cell lymphoma 2 (BCL-2) family inhibitors like navitoclax, remain the most widely used senolytics *in vivo*, showing promise across a spectrum of age-related conditions (211, 212). For instance, navitoclax clearance of senescent cells attenuates cardiac fibrosis and vascular remodeling (213). In addition, fisetin, a natural flavonoid, has also proven to be a potent senolytic, restoring tissue homeostasis and extending lifespan in aged mice (214). These first-generation senolytics validate the principle that removing senescent cells can produce broad therapeutic benefits.

The senolytic arsenal continues to expand with compounds such as venetoclax, luteolin, piperlongumine, and curcumin analogs (215–217). Furthermore, novel mechanisms for senescent cell targeting are emerging. These include antagonizing thrombomodulin-initiated signaling to alleviate liver fibrosis (218), using the HSP90 inhibitor XL888 to eliminate pathogenic p16^Ink4a+^ fibroblasts (219), and employing a trilocked photodynamic strategy for the immunogenic clearance of senescent cells in liver fibrosis (220). Cellular senescence is highly heterogeneous, with distinct subpopulations exhibiting opposing secretory phenotypes: a pro-inflammatory, senolytic-sensitive state versus a TGF-β-driven, pro-fibrotic and less inflammatory state that shows differential sensitivity to senolytics (221). This TGF-β-enriched subtype, characterized by extracellular matrix remodeling and reduced inflammation (222), underscores the plasticity of senescent cell fates and the need for context-dependent therapeutic strategies.

### 5.2 Senomorphics

In contrast to senolytics, senomorphics aim not to kill senescent cells but to suppress the development of the senescent phenotype or inhibit the production of the detrimental SASP. This class includes inhibitors of phosphoinositide-3-kinase (PI3K)-mechanistic signaling, novel antioxidants, and sirtuin activators (223). For example, the nucleoside reverse transcriptase inhibitor lamivudine reduces the late SASP and ameliorates aging phenotypes in mice (224). A major focus is the cyclic GMP-AMP synthase-stimulator of interferon genes (cGAS-STING) pathway, a primordial immune sensor now recognized as a critical regulator of senescence and organ fibrosis (225, 226). Genetic or pharmacological inhibition of cGAS or STING suppresses the SASP and reduces tissue inflammation and fibrosis (227–229), linking it to a range of chronic diseases, such as fibrosis and auto-inflammatory diseases (27). Similarly, the NLRP3 inflammasome is a key SASP regulator, making it an active drug development target (230). Targeting these innate immune signaling hubs represents a strategic approach to dampen the pervasive inflammation fueled by the SASP.

Other senomorphic strategies involve modulating the transcriptional regulation of the SASP. The JAK1/2 inhibitor ruxolitinib reduces proinflammatory SASP factors and frailty in aging mice (231) and reprograms the SASP to foster anti-tumor immunity (232). Epigenetic modifiers, such as the BRD4 inhibitor IBET762 (233) and the histone deacetylase (HDAC) inhibitor Trichostatin A (234), also effectively suppress SASP expression. Established drugs like glucocorticoids and metformin exert part of their beneficial effects by inhibiting NF-κB and thus blunting the proinflammatory arm of the SASP (235, 236). Additional agents with senomorphic properties include cardiac glycosides, which target the Na^+^/K^+^ ATPase pump in senescent cells to alleviate lung fibrosis (237), and molecules like nephronectin (238) or peptidomimetic telomere dysfunction inhibitors (239) that enhance tissue resistance to senescence and fibrosis. The RNA-binding protein YTHDC1 has also been identified as an m6A-independent regulator that delays cellular senescence and pulmonary fibrosis (240). The breadth of senomorphic targets underscores the complexity of the senescence program while offering multiple avenues for therapeutic intervention without requiring cell death.

### 5.3 Immune-mediated clearance of senescent cells

Harnessing and enhancing the immune system’s innate capacity to clear senescent cells is a rapidly advancing frontier. Chimeric antigen receptor (CAR) T cell therapy has shown pre-clinical promise in targeting senescent cells (241–244). Furthermore, specific cytotoxic immune populations play natural roles: Cytotoxic CD4^+^T cells can eliminate senescent cells via recognition of cytomegalovirus antigens (245), and cytotoxic T lymphocyte-associated protein 4 (CTLA4)-expressing CD8^+^T cells from fibrotic mice exhibit enhanced cytolytic activity against senescent cells (246). The immune clearance process can be influenced by senescent cell-intrinsic factors. For example, CDK inhibitor p21 (CDKN1A) not only regulates senescence but also controls ECM component expression in fibrotic lung senescent cells, affecting their microenvironment (247, 248). These findings highlight the dynamic interplay between senescent cells and the immune system, which can be therapeutically co-opted.

Novel delivery and targeting systems are being developed to precision-engineer immune-mediated clearance. Examples include using a gene-editable FePt dual-atom catalyst to specifically target senescent AEC2 in pulmonary fibrosis (249). Other strategies focus on modulating key pathways within tissues to reduce the senescent burden, such as targeting the non-canonical cGAS-STING pathway (225) or knocking down neuraminidase 4 in the kidney to diminish pro-fibrotic cytokines and senescence (250). Conversely, protective immune mechanisms exist, as demonstrated by CXCR6 promoting NKT- and CD4^+^T-cell-dependent control of senescence to inhibit liver cancer (251). Importantly, disrupting beneficial senescence programs can be detrimental, as ablation of a p53-dependent senescence pathway in hepatic stellate cells exacerbates liver fibrosis (252). This underscores the need for precise, context-specific strategies that selectively eliminate or modulate pathogenic senescent cells while sparing those involved in physiological repair.

## 6 Conclusion

Cellular senescence constitutes a double−edged sword in the pathogenesis of intestinal fibrosis in CD. Acute senescence may aid in wound healing and limit excessive fibrogenesis, whereas chronic persistence of senescent cells-through sustained SASP secretion-drives inflammation, ECM remodeling, and irreversible stricture formation. The CD microenvironment accelerates senescence across multiple cell lineages, creating a self−amplifying loop that perpetuates fibrosis.

Emerging therapeutic strategies aimed at the selective elimination of senescent cells (senolytics), the modulation of their secretory phenotype (senomorphics), or approaches that enhance immune-mediated clearance, hold considerable promise. However, key challenges remain: (1) identifying specific surface markers for intestinal senescent cells to enable targeted therapy; (2) determining the optimal timing of intervention to avoid disrupting beneficial senescence; (3) understanding organ− and context−specific differences in senescence pathways; and (4) translating preclinical findings into safe and effective clinical trials for CD patients.

Future research should focus on single−cell and spatial transcriptomics to delineate senescent cell heterogeneity in CD strictures, develop senescence−imaging biomarkers, and design combinatorial approaches that integrate senescence−targeting agents with existing anti−inflammatory therapies. Ultimately, a nuanced understanding of senescence in CD fibrosis will unlock novel avenues to prevent, halt, or even reverse intestinal strictures, thereby improving long−term outcomes for patients.
